# Psoriasis and type 2 diabetes mellitus: epidemiology, shared mechanisms, and therapeutic implications: a narrative review

**DOI:** 10.3389/fmed.2026.1897186

**Published:** 2026-08-10

**Authors:** Amr Molla

**Affiliations:** Department of Medicine, College of Medicine, Taibah University, Madinah, Saudi Arabia

**Keywords:** biologics, chronic inflammation, insulin resistance, metabolic comorbidity, psoriasis, type 2 diabetes mellitus

## Abstract

Psoriasis is a chronic immune-mediated inflammatory disorder increasingly recognized as a systemic disease rather than an isolated cutaneous condition. In addition to its dermatologic burden, psoriasis is associated with multiple metabolic comorbidities, among which type 2 diabetes mellitus (T2DM) is particularly important because of its prevalence, long-term complications, and potential bidirectional relationship with psoriatic inflammation. Epidemiologic studies consistently suggest that patients with psoriasis, especially those with moderate-to-severe or longstanding disease, have a higher prevalence and incidence of T2DM than the general population. Conversely, metabolic dysfunction, particularly insulin resistance and obesity-related inflammation, may contribute to psoriasis onset and severity. Shared pathogenic pathways include chronic systemic inflammation, dysregulated adipokines, endothelial dysfunction, oxidative stress, and overlapping genetic susceptibility. These mechanistic links have important clinical implications for screening, cardiovascular risk assessment, and therapeutic decision-making. Emerging evidence also suggests that some antidiabetic agents and biologic therapies may influence both metabolic and dermatologic outcomes, although current interventional evidence remains limited and heterogeneous. This narrative review summarizes the epidemiologic evidence supporting the psoriasis–T2DM association, examines shared inflammatory and metabolic mechanisms, and discusses the clinical and therapeutic implications of this comorbidity. Recognizing psoriasis as a systemic inflammatory disease with substantial metabolic consequences may support earlier risk stratification, multidisciplinary care, and more individualized treatment strategies.

## 1 Introduction

Psoriasis is a chronic immune-mediated inflammatory disease that primarily affects the skin but is now widely regarded as a systemic disorder with important extracutaneous manifestations (1, 2). It affects approximately 2–4% of the global population, although prevalence varies by geographic region, ethnicity, and methodology of case ascertainment (3, 4). The disease may occur at any age and often demonstrates a bimodal age distribution, with peaks in early adulthood and later life (5). Although most patients have mild disease, a clinically important subset develops moderate-to-severe psoriasis, which is more strongly linked to systemic inflammation and comorbidity burden (1, 2).

Over the past two decades, psoriasis has been increasingly associated with cardiometabolic disorders, including T2DM and metabolic syndrome, the latter encompassing central obesity, hypertension, dyslipidemia, and impaired glucose regulation (6–8). This association is clinically significant because these comorbidities contribute substantially to long-term morbidity, mortality, treatment complexity, and healthcare utilization. Meta-analytic data indicate that diabetes is more common among patients with psoriasis than among individuals without psoriasis, particularly in those with more severe disease or concomitant psoriatic arthritis (6, 8).

T2DM itself represents a major global public health challenge. Current international estimates indicate that hundreds of millions of adults are living with diabetes worldwide, with prevalence continuing to increase because of aging populations, sedentary lifestyles, and the global rise in obesity (9). T2DM is associated with microvascular and macrovascular complications, including nephropathy, neuropathy, retinopathy, and cardiovascular disease. From a dermatologic standpoint, diabetes is also relevant because it contributes to impaired wound healing, increased susceptibility to infection, and altered inflammatory responses (9, 10).

A growing body of epidemiologic evidence suggests that psoriasis is associated with an increased risk of incident T2DM, independent of several conventional risk factors (7, 11–13). The relationship appears to be influenced by psoriasis severity, disease duration, and the presence of psoriatic arthritis, supporting the concept that cumulative systemic inflammatory burden may contribute to metabolic dysfunction (6, 14–16). At the same time, emerging data support the possibility of bidirectionality, whereby insulin resistance and metabolic inflammation may worsen psoriasis severity or promote disease expression (17, 18).

Several mechanisms may explain this association. Psoriasis and T2DM share chronic low-grade inflammation, cytokine dysregulation, adipokine imbalance, oxidative stress, endothelial dysfunction, and, at least in part, overlapping genetic susceptibility (17–19). These common pathways provide a plausible biological basis for the epidemiologic link and have important implications for cardiovascular risk, screening practices, and therapeutic decision-making.

Despite this progress, important gaps remain. Existing studies vary in design, patient populations, severity definitions, and control for confounding factors such as obesity, smoking, and physical activity. In addition, interventional data remain limited regarding whether effective treatment of psoriasis modifies diabetes risk or improves metabolic outcomes (10, 17, 19). Accordingly, this narrative review aims to summarize the epidemiologic evidence supporting the association between psoriasis and T2DM, examine the shared inflammatory and metabolic mechanisms underlying this relationship, and discuss the clinical and therapeutic implications for integrated patient care.

## 2 Materials and methods

This article is a narrative review designed to synthesize current evidence regarding the epidemiologic association, potential bidirectional relationship, shared pathophysiological mechanisms, and clinical implications linking psoriasis and T2DM. A narrative design was selected because the literature includes heterogeneous study types, including observational cohorts, meta-analyses, mechanistic studies, and therapeutic reports, which are not readily amenable to a single quantitative synthesis.

### 2.1 Information sources and search strategy

Relevant literature was identified through searches of PubMed, Scopus, and Google Scholar from database inception to January 31, 2026. The search strategy used combinations of controlled vocabulary and free-text terms related to psoriasis, type 2 diabetes mellitus, insulin resistance, metabolic syndrome, systemic inflammation, cardiovascular risk, biologic therapy, GLP-1 receptor agonists, DPP-4 inhibitors, and antidiabetic agents. Search terms included combinations of “psoriasis,” “type 2 diabetes mellitus,” “T2DM,” “diabetes,” “insulin resistance,” “metabolic syndrome,” “obesity,” “systemic inflammation,” “cardiovascular risk,” “biologics,” “TNF inhibitor,” “IL-17 inhibitor,” “IL-23 inhibitor,” “GLP-1 receptor agonists,” “DPP-4 inhibitors,” and “antidiabetic drugs.” Reference lists of key reviews, meta-analyses, and major epidemiologic studies were also manually screened to identify additional relevant publications.

### 2.2 Eligibility and study selection

Eligible publications included observational cohort studies, case–control studies, cross-sectional studies, meta-analyses, systematic reviews, mechanistic studies, genetic studies, and clinically relevant therapeutic reports addressing one or more of the following themes: epidemiologic association between psoriasis and type 2 diabetes mellitus, temporal or bidirectional relationship, psoriasis severity or phenotype in relation to metabolic risk, shared inflammatory or metabolic mechanisms, cardiovascular implications, or therapeutic interactions between antidiabetic and antipsoriatic treatments. Studies focused exclusively on type 1 diabetes mellitus, gestational diabetes, or unrelated diabetes outcomes were not prioritized. Case reports were considered only in selected therapeutic contexts when higher-level evidence was limited or when they illustrated clinically relevant safety issues.

Because this was a narrative review rather than a systematic review, studies were not selected according to a formal risk-of-bias scoring system or quantitative meta-analytic protocol. Instead, publications were prioritized qualitatively according to relevance to the review objectives, study design, sample size, recency, adjustment for important confounders, clinical applicability, and contribution to mechanistic or therapeutic understanding. Greater emphasis was placed on systematic reviews, meta-analyses, large population-based cohorts, prospective studies, and recent clinically relevant reviews. Literature screening, study selection, interpretation, and narrative synthesis were performed by the sole author.

### 2.3 Data synthesis

Evidence was synthesized qualitatively and organized into the following domains: epidemiologic association, bidirectional relationship, shared pathophysiological mechanisms, cardiovascular and clinical implications, therapeutic interactions, and limitations of current evidence. Particular attention was given to studies that evaluated psoriasis severity, disease duration, psoriatic arthritis, obesity, cardiometabolic comorbidity, treatment exposure, or adjustment for conventional diabetes risk factors. Conflicting findings were interpreted cautiously with attention to differences in study design, psoriasis case definitions, severity classification, population characteristics, ascertainment of diabetes, and residual confounding. No formal pooled effect estimate was calculated because the review was designed as a narrative synthesis and the included evidence was heterogeneous in design, outcomes, and analytic methods.

## 3 Epidemiologic evidence linking psoriasis and type 2 diabetes mellitus

Multiple epidemiologic studies have shown that psoriasis is associated with an increased risk of T2DM (6, 8, 11, 16). Meta-analyses and systematic reviews have generally reported a modest but statistically significant increase in diabetes prevalence and incidence among patients with psoriasis, with effect estimates often ranging from approximately 1.2 to 1.5 depending on study design and population (7, 8). These findings support the concept that psoriasis is more than a skin-limited disease and that its systemic inflammatory component may contribute to metabolic risk.

Large cohort studies from the United States, Denmark, and Taiwan have reported higher rates of incident T2DM in patients with psoriasis, even after adjustment for conventional risk factors such as age, body mass index, smoking, and other comorbidities (10, 11, 16). The magnitude of risk appears greater among patients with moderate-to-severe psoriasis, suggesting a severity-dependent relationship (7, 14, 16). This observation is biologically plausible because greater disease severity is generally associated with higher inflammatory burden, more extensive immune activation, and more pronounced metabolic dysregulation. A summary of representative epidemiologic studies is provided in Table 1.

**Table 1:** Key epidemiologic studies on the association between psoriasis and T2DM.

First author, year	Country/population	Study design	Main focus	Key finding	Clinical relevance
Qureshi et al., 2009 (11)	United States, female nurses	Prospective cohort study	Psoriasis and incident diabetes/hypertension	Psoriasis was associated with a higher risk of diabetes and hypertension in women	Supports psoriasis as a systemic disease with metabolic consequences
Li et al., 2012 (12)	United States, women and men	Cohort study	Psoriasis and risk of T2DM	Psoriasis was associated with increased risk of T2DM in both sexes	Strengthens the epidemiologic link across broader populations
Khalid et al., 2013 (13)	Denmark, nationwide population	Nationwide cohort study	Psoriasis and new-onset diabetes	Patients with psoriasis had higher risk of developing new-onset diabetes	Provides large-scale real-world evidence
Lee et al., 2014 (14)	Taiwan, nationwide population	Population-based cohort study	Severity of psoriasis and diabetes risk	Diabetes risk increased with psoriasis severity and concomitant medication burden	Suggests a severity-dependent association
Wan et al., 2018 (15)	Population-based cohort	Prospective cohort study	Psoriasis and incident diabetes	Psoriasis was associated with a higher future risk of diabetes	Reinforces longitudinal relationship
	Denmark, nationwide population	Nationwide cohort study	Psoriasis and diabetes risk	Psoriasis was associated with increased diabetes risk in a large national dataset	Supports reproducibility across healthcare systems
Armstrong et al., 2013 (7)	Multiple populations	Systematic review and meta-analysis	Psoriasis and diabetes risk	Meta-analysis showed a significantly increased risk of diabetes among patients with psoriasis	High-level summary evidence for association
Mamizadeh et al., 2019 (8)	Multiple populations	Systematic review and meta-analysis	Psoriasis and diabetes mellitus	Confirmed an association between psoriasis and diabetes across pooled studies	Supports consistency of epidemiologic signal
Coto-Segura et al., 2013 (6)	Multiple studies	Systematic review	Psoriasis, psoriatic arthritis, and T2DM	T2DM was more frequent in psoriasis/psoriatic arthritis, especially with greater inflammatory burden	Highlights importance of disease duration and systemic involvement
Takeshita et al., 2017 (16)	Review of epidemiologic literature	Narrative epidemiologic review	Psoriasis comorbid diseases	Summarized broad evidence linking psoriasis with metabolic and cardiovascular comorbidities	Useful for contextualizing psoriasis within multimorbidity

Disease duration also appears clinically relevant. Patients with longstanding psoriasis and those with psoriatic arthritis may have higher cumulative metabolic risk than those with limited or short-duration disease (6, 10–13). Persistent systemic inflammation may contribute over time to insulin resistance, endothelial dysfunction, and related metabolic abnormalities. In addition, patients with psoriasis frequently have coexisting obesity, dyslipidemia, hypertension, and metabolic syndrome, which may further amplify diabetes risk (10, 16, 20).

Not all studies have reported identical results, and some population-based analyses have suggested weaker or nonsignificant associations after extensive adjustment for confounders (8, 21, 22). Nevertheless, the overall body of evidence supports a clinically meaningful relationship between psoriasis and T2DM, particularly in patients with more severe disease or higher cardiometabolic burden.

Several sources of uncertainty should be considered when interpreting this epidemiologic association. First, psoriasis and T2DM share several common risk factors, particularly obesity, sedentary lifestyle, smoking, dyslipidemia, hypertension, and socioeconomic determinants of health. Although many cohort studies adjust for some of these variables, residual confounding remains difficult to exclude fully, especially when body mass index, central adiposity, physical activity, dietary patterns, and medication exposure are incompletely captured. Second, studies vary in how psoriasis is defined, with some relying on diagnostic codes, prescription records, hospital-based cohorts, or self-reported disease, while others use severity proxies such as systemic treatment or phototherapy. These differences may influence risk estimates and partly explain why some studies report stronger associations than others. Third, diabetes ascertainment also varies across studies, including physician diagnosis, medication use, laboratory criteria, or registry-based definitions. Therefore, the observed association should be interpreted as clinically meaningful but not fully causal. The strongest evidence supports increased metabolic risk among patients with more severe, longstanding, or systemic psoriasis, but further prospective studies with rigorous phenotyping and better control of lifestyle and adiposity-related confounders are still needed.

The relationship between clinical psoriasis subtypes and T2DM risk remains less clearly defined than the relationship between psoriasis severity and metabolic risk. Most epidemiologic studies classify psoriasis broadly or use severity proxies such as systemic therapy, phototherapy, hospitalization, or psoriatic arthritis rather than detailed clinical morphology (23). Therefore, it remains uncertain whether plaque, guttate, inverse, pustular, erythrodermic, nail, or scalp-predominant psoriasis differ independently in their association with T2DM after adjustment for severity, obesity, systemic inflammation, and treatment exposure. Nevertheless, subtype classification may have practical relevance (24). Chronic plaque psoriasis accounts for most available evidence and is the phenotype most commonly linked to cardiometabolic comorbidity in population-based studies. Pustular and erythrodermic psoriasis, although less common, may reflect a higher systemic inflammatory burden and often require systemic therapy (6), which may justify closer metabolic assessment. Inverse psoriasis may be more frequent or more difficult to manage in patients with obesity and insulin resistance, although whether it independently predicts T2DM remains uncertain. Psoriatic arthritis may be particularly relevant because it represents systemic musculoskeletal inflammation and has been associated with higher comorbidity burden (6). Clinically, these distinctions suggest that dermatologists should consider not only psoriasis extent and severity, but also phenotype, inflammatory burden, treatment history, and associated psoriatic arthritis when assessing cardiometabolic risk.

## 4 Bidirectional relationship and shared pathophysiological mechanisms

The association between psoriasis and T2DM is increasingly viewed as bidirectional rather than unidirectional alone (17, 18). On one hand, psoriatic inflammation may promote insulin resistance and metabolic dysfunction. On the other, preexisting metabolic abnormalities, particularly obesity-related inflammation and impaired glucose homeostasis, may exacerbate psoriatic activity or contribute to disease onset in susceptible individuals (17, 19).

A central mechanism linking the two conditions is chronic systemic inflammation. Psoriasis is characterized by dysregulated innate and adaptive immune responses, with major roles for tumor necrosis factor-alpha (TNF-α), interleukin-17 (IL-17), interleukin-23 (IL-23), and interleukin-6 (IL-6) (1, 2). These same inflammatory mediators are also implicated in insulin resistance, endothelial injury, and metabolic stress (2, 17, 19). Persistent exposure to pro-inflammatory cytokines may impair insulin signaling pathways in peripheral tissues, promote hepatic glucose dysregulation, and contribute to pancreatic beta-cell dysfunction.

Adipose tissue biology is also important. Obesity, which is common among patients with psoriasis, is associated with secretion of pro-inflammatory adipokines such as leptin and reduced levels of anti-inflammatory adiponectin (17, 18, 25). These alterations may amplify both systemic inflammation and insulin resistance. In psoriasis, this inflammatory-metabolic environment may further support keratinocyte hyperproliferation, T-cell activation, and maintenance of chronic plaque inflammation.

Oxidative stress and endothelial dysfunction provide additional mechanistic overlap (2, 18, 20, 25). Patients with psoriasis demonstrate evidence of vascular inflammation and metabolic perturbation that extend beyond the skin. These abnormalities may contribute to accelerated atherosclerosis, impaired insulin sensitivity, and increased cardiovascular risk. The concept of the “psoriatic march” proposes that chronic cutaneous and systemic inflammation in psoriasis may drive insulin resistance, endothelial dysfunction, and ultimately cardiovascular morbidity (20, 26).

Genetic studies have also suggested partial overlap between psoriasis susceptibility and metabolic disease pathways (19). A trans-disease meta-analysis evaluating psoriasis and T2DM identified shared loci with evidence of colocalization and concordant direction of effect, including loci related to ACTR2, ERLIN1, TRMT112, and BECN1, with functional connections to NF-κB signaling (19). This is biologically relevant because NF-κB is involved in inflammatory cytokine production, immune-cell activation, adipose tissue inflammation, and insulin resistance. In addition, established psoriasis susceptibility loci involve immune pathways that may intersect with metabolic inflammation, including HLA-C, IL23A, IL23R, IL12B, TNIP1, TNFAIP3, and NFKBIZ (27). These genes are linked to antigen presentation, IL-23/Th17 signaling, TNF-related signaling, and NF-κB regulation, which are central to psoriatic inflammation and may also contribute to systemic inflammatory states that impair insulin signaling. Although these genetic findings do not fully explain the clinical association between psoriasis and T2DM, they support the concept that shared immunometabolic pathways may predispose some patients to both cutaneous inflammation and metabolic dysfunction. These overlapping mechanisms and their clinical implications are summarized in Table 2.

**Table 2:** Shared pathophysiological mechanisms and clinical implications linking psoriasis and T2DM.

Mechanism	Role in psoriasis	Role in T2DM	Clinical implication
Chronic systemic inflammation	Sustains keratinocyte proliferation and immune activation	Promotes insulin resistance and metabolic dysfunction	Supports viewing psoriasis as a systemic inflammatory disorder
TNF-α	Major pro-inflammatory cytokine in psoriatic plaques	Impairs insulin signaling and contributes to metabolic stress	Explains overlap between psoriasis activity and metabolic risk
IL-17/IL-23 axis	Central pathway in psoriatic pathogenesis	Linked to chronic inflammation and insulin resistance	May partially explain shared immunometabolic pathways
IL-6	Contributes to inflammatory amplification	Associated with insulin resistance and endothelial injury	Connects inflammatory burden to metabolic and vascular complications
Insulin resistance	May worsen inflammatory skin disease	Core feature of T2DM	Supports bidirectionality between metabolic dysfunction and psoriasis severity
Adipokine dysregulation (e.g., leptin, adiponectin)	Favors persistent inflammation in obesity-associated psoriasis	Contributes to glucose dysregulation and insulin resistance	Highlights the importance of obesity and weight management
Oxidative stress	Enhances inflammatory signaling and tissue damage	Worsens beta-cell stress and vascular dysfunction	Supports common downstream injury pathways
Endothelial dysfunction	Reflects systemic vascular involvement in psoriasis	Contributes to atherosclerosis and diabetic vascular complications	Helps explain increased cardiovascular risk in comorbid patients
Obesity/metabolic syndrome	Increases psoriasis severity and treatment complexity	Strongly associated with T2DM development	Major confounder and therapeutic target
Shared genetic susceptibility	Some loci overlap with inflammatory pathways	Some loci overlap with metabolic disease susceptibility	Suggests a biologic predisposition to both conditions in some patients
Psoriatic march	Chronic inflammation progresses to insulin resistance and vascular disease	Integrates metabolic and cardiovascular consequences	Useful conceptual framework for risk stratification

## 5 Cardiovascular and clinical implications

The coexistence of psoriasis and T2DM has important clinical consequences because both disorders independently increase cardiovascular risk (10, 20, 26). When present together, they may contribute synergistically to endothelial dysfunction, accelerated atherosclerosis, and major adverse cardiovascular outcomes. This cumulative burden is particularly relevant in patients with severe psoriasis, longstanding disease, obesity, hypertension, dyslipidemia, or smoking history.

From a practical standpoint, clinicians should recognize psoriasis as a systemic inflammatory disease that warrants broader cardiometabolic assessment, especially in patients with moderate-to-severe disease or additional risk factors (10, 25). Screening for diabetes, obesity, blood pressure abnormalities, dyslipidemia, and other metabolic disturbances should be considered as part of routine comprehensive care. Early identification of metabolic abnormalities may allow timely intervention through lifestyle modification, glycemic control, weight management, and cardiovascular risk reduction.

A multidisciplinary approach is often appropriate. Collaboration among dermatologists, primary care physicians, endocrinologists, and, when necessary, cardiologists may improve risk stratification and long-term outcomes (10, 25). Patient education is also important because awareness of psoriasis-related metabolic risk remains variable, and treatment adherence may be influenced by the burden of comorbid disease.

## 6 Therapeutic implications

### 6.1 Antidiabetic agents with potential dermatologic benefit

Because psoriasis and T2DM share inflammatory and metabolic pathways, some antidiabetic agents have been explored for possible dermatologic benefit.

#### 6.1.1 Thiazolidinediones and metformin

The strongest antidiabetic evidence for direct psoriasis improvement has historically involved thiazolidinediones, particularly pioglitazone. Small randomized and nonrandomized studies have suggested improvement in psoriasis severity, including reductions in Psoriasis Area and Severity Index (PASI) scores, possibly through effects on insulin sensitivity and inflammatory signaling (28, 29). However, the evidence base remains limited, and concerns regarding adverse effects and broader safety considerations restrict enthusiasm for routine dermatologic use.

Metformin has also been discussed as a potentially relevant agent because of its favorable metabolic profile and anti-inflammatory effects, particularly in obese or insulin-resistant patients with psoriasis (10, 28, 29). Nonetheless, direct evidence for clinically meaningful psoriasis improvement with metformin is less robust than for glycemic benefit, and current data do not justify presenting it as an established antipsoriatic therapy.

#### 6.1.2 Glucagon-like peptide-1 (GLP-1) receptor agonists

GLP-1 receptor agonists have received increasing attention because of their favorable effects on body weight, glycemic control, and cardiovascular outcomes, all of which are highly relevant to psoriatic patients with metabolic comorbidity (10, 30). Case reports and small observational studies have suggested possible improvement in psoriasis severity or quality-of-life measures in selected patients, particularly those with obesity or T2DM (30). Proposed mechanisms include immunometabolic modulation, reduction of systemic inflammatory activity, and indirect benefit mediated through weight loss and improved insulin sensitivity.

However, the dermatologic evidence for GLP-1 receptor agonists remains emerging rather than established. Available data include case reports, small observational studies, limited prospective cohorts, and a small number of interventional studies with heterogeneous findings (30). Some reports suggest improvement in PASI scores or dermatology-related quality-of-life measures, particularly among patients with obesity or T2DM, whereas other studies have not demonstrated consistent short-term cutaneous benefit. Therefore, GLP-1 receptor agonists should not currently be regarded as primary antipsoriatic agents. Their strongest evidence-based role remains metabolic and cardiovascular risk reduction in appropriately selected patients with T2DM, obesity, or related cardiometabolic indications. Any dermatologic improvement should be interpreted as a potential adjunctive or indirect benefit mediated through weight loss, improved insulin sensitivity, and reduced systemic inflammatory burden rather than as a proven psoriasis-specific therapeutic effect.

#### 6.1.3 Dipeptidyl peptidase-4 (DPP-4) inhibitors

Recent observational evidence has suggested that DPP-4 inhibitors may be associated with lower psoriasis risk in patients with T2DM compared with some alternative glucose-lowering therapies (31). Proposed mechanisms include effects on keratinocyte biology and local immune regulation. However, this evidence is preliminary, derived largely from pharmacoepidemiologic data, and does not establish a therapeutic role in existing psoriasis. At present, DPP-4 inhibitors may be considered metabolically reasonable options in selected diabetic patients, but their dermatologic benefit remains investigational.

### 6.2 Psoriasis biologics and metabolic outcomes

Because TNF-α, IL-17, IL-23, and related inflammatory pathways contribute to both psoriatic inflammation and metabolic dysfunction, biologic therapies have been hypothesized to favorably influence insulin resistance or glycemic outcomes (2, 17, 28). However, clinical evidence remains inconsistent. Some small studies and observational reports have suggested modest improvement in metabolic markers or glycated hemoglobin in selected patients after biologic treatment, whereas more recent cardiometabolic studies have shown that substantial improvement in skin severity does not necessarily translate into significant improvement in HbA1c or lipid parameters (28, 32). These findings suggest that systemic inflammatory control may be biologically relevant to metabolic risk, but biologic agents should not be considered diabetes-modifying therapies on the basis of current evidence. Large prospective studies and interventional trials with predefined metabolic endpoints are still needed to determine whether specific biologic classes differ in their effects on insulin resistance, incident diabetes, or long-term cardiometabolic outcomes.

Case reports have also described clinically important hypoglycemia in diabetic patients after initiation of TNF-α inhibitors, presumably because of rapid improvement in insulin sensitivity (33). Although such reports are uncommon and cannot define incidence, they highlight a practical point: in patients receiving insulin or insulin secretagogues, metabolic parameters should be monitored carefully when major anti-inflammatory therapy is initiated. Overall, biologics should not currently be promoted as diabetes treatments, but their metabolic effects deserve clinical awareness and further investigation.

### 6.3 Conventional psoriasis therapies and metabolic risk

Traditional systemic psoriasis therapies may also influence metabolic risk, either directly or indirectly (10, 28, 34). Methotrexate has complex effects; although some observational data have raised concern about diabetes risk under certain exposure conditions, methotrexate may also confer anti-inflammatory cardiovascular benefit in some patient populations (34). The net metabolic effect likely depends on patient selection, cumulative exposure, and comorbidity profile.

Phototherapy, particularly psoralen plus ultraviolet A (PUVA), has been associated in observational work with increased diabetes risk, though this may partly reflect confounding by indication because patients receiving PUVA often have more extensive or severe psoriasis (34). Cyclosporine, meanwhile, has recognized metabolic drawbacks, including effects on blood pressure, renal function, and potentially glucose regulation, although direct diabetes-specific evidence remains limited (10, 28, 34).

In addition, commonly prescribed non-dermatologic medications may modify risk. For example, thiazide diuretics, often used in patients with hypertension, may contribute to worsening glycemic parameters in predisposed patients (34). These issues reinforce the need to individualize therapy in psoriatic patients with metabolic disease.

### 6.4 Practical clinical management

In clinical practice, these findings support a risk-stratified approach rather than universal extensive testing for all patients with psoriasis. Patients with moderate-to-severe psoriasis, longstanding disease, psoriatic arthritis, obesity, family history of diabetes, hypertension, dyslipidemia, or other features of metabolic syndrome may warrant more systematic cardiometabolic assessment. This may include periodic measurement of body mass index or waist circumference, blood pressure, fasting glucose or glycated hemoglobin, and lipid profile, in coordination with primary care or endocrinology when abnormalities are detected (10). Dermatologists should also consider metabolic comorbidity when selecting systemic therapy, because obesity, diabetes, hepatic steatosis, renal disease, cardiovascular disease, and concomitant medications may influence treatment efficacy, safety, and monitoring requirements. Importantly, the purpose of screening is not only to identify diabetes but also to recognize broader cardiometabolic risk and to encourage earlier lifestyle and pharmacologic intervention when clinically indicated (25).

## 7 Limitations of current evidence and future research directions

Despite substantial progress, several limitations affect the current literature. First, much of the epidemiologic evidence is observational, which limits causal inference. Residual confounding by obesity, smoking, diet, physical activity, healthcare utilization, and socioeconomic factors remains difficult to eliminate fully (8, 17, 21). Second, study heterogeneity is considerable, particularly regarding psoriasis severity classification, disease duration, case definitions, and adjustment strategies (7, 16, 17).

Third, although several mechanistic links have been proposed, the precise molecular pathways connecting psoriasis and T2DM remain incompletely defined (17, 19, 25). It is likely that immune dysregulation, adipose inflammation, oxidative stress, endothelial dysfunction, and genetic susceptibility interact in a complex and dynamic manner rather than through a single dominant pathway.

Fourth, interventional evidence is limited. Most data regarding the metabolic effects of psoriasis biologics or the dermatologic effects of antidiabetic medications derive from case reports, small cohorts, secondary analyses, or narrative reviews rather than adequately powered randomized trials (28, 30, 31, 33). This is particularly relevant for GLP-1 receptor agonists and biologic therapies, where enthusiasm has outpaced definitive evidence.

Future research should prioritize large prospective cohorts with rigorous phenotyping and better control of confounding factors. Studies should clarify whether psoriasis independently increases diabetes risk beyond shared metabolic predisposition, and whether risk differs meaningfully by phenotype, severity, duration, or treatment exposure. Dedicated mechanistic studies are needed to delineate the immunometabolic pathways linking both disorders. Finally, well-designed interventional trials should evaluate whether specific psoriasis therapies improve metabolic outcomes, and whether selected antidiabetic agents offer reproducible dermatologic benefit in patients with comorbid disease.

## 8 Conclusion

Psoriasis and T2DM are interconnected chronic disorders linked by shared inflammatory, metabolic, and vascular pathways. Epidemiologic evidence supports an increased burden of diabetes among patients with psoriasis, particularly in those with more severe, longstanding, or systemic disease. In parallel, insulin resistance and metabolic inflammation may contribute to psoriasis onset or severity, supporting a bidirectional relationship. These observations reinforce the concept of psoriasis as a systemic inflammatory disease with important cardiometabolic implications. Clinically, this relationship supports earlier screening, broader cardiovascular risk assessment, and more integrated multidisciplinary care. Although some therapeutic overlap is promising, particularly in the context of modern biologics and selected antidiabetic agents, current evidence remains insufficient to support strong therapeutic claims. Further high-quality mechanistic and interventional research is needed to define how best to reduce both dermatologic and metabolic risk in this patient population.
